# TRP Channels as Sensors of Chemically-Induced Changes in Cell Membrane Mechanical Properties

**DOI:** 10.3390/ijms20020371

**Published:** 2019-01-16

**Authors:** Justyna B. Startek, Brett Boonen, Karel Talavera, Victor Meseguer

**Affiliations:** 1 Laboratory of Ion Channel Research, Department of Cellular and Molecular Medicine, KU Leuven; VIB Center for Brain & Disease Research, Herestraat 49, Campus Gasthuisberg O&N1 bus 802, 3000 Leuven, Belgium; justyna.startek@kuleuven.vib.be (J.B.S.); brett.boonen@kuleuven.vib.be (B.B.); 2 Instituto de Neurociencias de Alicante, Universidad Miguel Hernández y CSIC, E-03550 Alicante , Spain; vmeseguer@umh.es

**Keywords:** cellular membranes, mechanosensation, TRP channels, LPS, lipophilic compounds

## Abstract

Transient Receptor Potential ion channels (TRPs) have been described as polymodal sensors, being responsible for transducing a wide variety of stimuli, and being involved in sensory functions such as chemosensation, thermosensation, mechanosensation, and photosensation. Mechanical and chemical stresses exerted on the membrane can be transduced by specialized proteins into meaningful intracellular biochemical signaling, resulting in physiological changes. Of particular interest are compounds that can change the local physical properties of the membrane, thereby affecting nearby proteins, such as TRP channels, which are highly sensitive to the membrane environment. In this review, we provide an overview of the current knowledge of TRP channel activation as a result of changes in the membrane properties induced by amphipathic structural lipidic components such as cholesterol and diacylglycerol, and by exogenous amphipathic bacterial endotoxins.

## 1. TRP Channels and Mechanosensitivity

Detecting and responding to mechanical stimuli such as thermal molecular agitation or osmotic pressure gradients is an indispensable part of any living system [1]. Many cells such as chondrocytes, endothelial and cochlear cells are continuously exposed to and regulated by mechanical stimuli such as shear and compressive forces [2,3,4]. As such, multiple proteins transduce mechanical stimuli exerted onto the membrane into an electrical and/or biochemical signal [5,6]. Mechanosensitive ion channels (MSCs) are a diverse population of proteins that can be divided into two categories. First, channels that respond to membrane tension due to the presence of specialized mechanosensing motifs/mechanical sensors or because their structure renders them susceptible to membrane tension [7]. Second, channels activated by stretch because the gating domain is intrinsically sensitive to membrane tension [8,9]. MSCs are fast transducers of mechanical stress and serve as both signaling sensors and effectors. They modify the electrical potential of cells and mediate the influx of specific ions, such as Ca^2+^, across the plasma membrane [7]. MSCs are implicated in a myriad of physiological processes, for example touch and pain sensation, hearing, blood pressure control and cell volume regulation [10].

Transient Receptor Potential channels (TRPs) are membrane-spanning proteins that form non-selective cation channels. TRP channels have been extensively studied and described as polymodal sensors, as they are responsible for transducing a wide variety of stimuli. TRPs are involved in chemosensation [11], thermosensation [12,13,14], mechanosensation [15] and photosensation [16,17]. For instance, several TRPs are highly sensitive to temperature changes and provide a broad thermosensitivity, extending from noxious cold to burning heat. How these channels are able to detect thermal stimuli remains a topic of investigation [12,18]. In addition, TRPs interact with a plethora of structurally unrelated exogenous and endogenous chemical ligands. In particular, some plant-derived chemicals favorably target the thermosensitive TRP channels, explaining why we attribute characteristic thermal traits with certain foods, such as a ‘cool’ mint and a ‘hot’ chili pepper [19,20,21]. In contrast to the huge number of exogenous compounds modulating TRPs, few endogenous ligands are known, yet the list is growing.

Nearly all cells in mammals express at least one member of the TRP channel family. Due to their widespread expression and localization, TRPs contribute to fundamental physiological processes, covering pure sensory functions (such as pheromone signaling, taste transduction, nociception and temperature sensation), homeostatic functions (such as Ca^2+^ and Mg^2+^ reabsorption and osmoregulation) and motile functions, such as muscle contraction and vasomotor control [13,22,23,24]. TRP channel activation leads to cation influx, membrane depolarization and the activation of Ca^2+^ dependent signaling pathways. Action potential firing in nociceptive sensory neurons generates the sensation of pain, while Ca^2+^ signaling activates intracellular pathways inducing altered membrane protein expression and triggering the release of signaling peptides [25,26].

The TRP channel family consists of 13 or 28 members depending on the species. Mammals contain 28 members divided in six subgroups according to amino acid sequence homology: TRPC (canonical), TRPV (vanilloid), TRPM (melastatin), TRPP (polycystin), TRPML (mucolipin) and TRPA (ankyrin). A seventh subgroup found in insects, nematodes, fish and amphibians is named TRPN, after the no-mechanoreceptor potential channel C (NOMP-C), a mechanotransduction channel required for gentle touch in *Drosophila* flies. For a detailed overview of the members of the different subgroups and their putative roles, we refer to other recent reviews and chapters [27,28]. Despite variations in sequence homology, TRP channels share a similar architecture. All TRP channels consist of identical or homologous tetramers. Each of the four subunits in the tetramer comprises six transmembrane (TM) domains, with intracellular carboxy and amino termini. TM5 and TM6 are linked by an extracellular loop and together they form the cation conduction pore.

TRP channels play a role in mechanotransduction pathways following fluid shear stress, and increased membrane tension and changes in cell volume or changes in osmolarity. The involvement of TRP channels in mechanosensation was discovered by a mutation in the *osm-9* gene of *Caenorhabditis elegans* that encodes a TRPV-like channel. This mutation led to defects in the avoidance to high osmolarity and nose touch [29]. Later, it was shown that TRPV1 is required for the response of osmosensory neurons in the organum vasculosum lamina terminalis, the primary osmosensor in the brain [30]. A deletion of 60 amino acids in the N-terminal region of TRPV1 renders stretch-inhibited cation channels [30,31,32]. In addition, multiple members of the TRPV subgroup are both mechano- and osmo-sensitive. TRPV2 functions as a mechanosensor in vascular smooth muscle cells and appears to be activated by osmotic cell swelling [33,34]. TRPV2 membrane insertion is promoted by PI3-kinase [35] and altered cellular PtdIns(4,5)P_2_ levels were proposed to underlie TRPV2 mechanosensitivity [36]. The interaction of TRPV2 with protein kinase A (PKA) suggest that PKA modification could modulate TRPV2 activity similarly as previously described for TRPV1 and the heat-activated K^+^ channel, TREK [37]. TRPV4 is another TRPV channel which is activated by osmotic cell swelling. While swelling induced activation of TRPV4 is independent of N-terminal ankyrin repeats [38], TRPV4 activation is mediated by the arachidonic acid metabolite 5′,6′-epoxyeicosatrienoic acid (5′,6′-EET) [39,40,41], consistent with the previously demonstrated swelling-induced activation of phospholipase A2 (PLA_2_) [42,43].

Multiple TRPC channels were identified as a mechanosensors, for example TRPC6 is activated by mechanical or osmotic stimuli that directly depends on the lateral-lipid tension and an energetic mismatch between protein and bilayer [44]. TRPC6 activity appears to be important for controlling the vascular tone in response to increased intravascular pressure [33,45,46].

Members of the TRPM subgroup also display putative mechanosensitive roles. Human TRPM3 channels display activity upon hypotonic cell swelling [47]. TRPM4 is involved in the control of pressure-induced smooth muscle cell depolarization, myogenic vasoconstriction in cerebral arteries and isolated vascular smooth muscle cells [45,48,49,50]. TRPM7 was proposed to be directly activated by cell stretch and potentiated by hypotonic cell swelling [51]. TRPM7 may also play a role in responses to shear stress, as its expression levels and responses are highly increased after stimulation [52].

TRPA1 has been proposed to function as a mechanosensor, but despite a large number of studies, its role in mechanosensitivity remains contentious. At first, mechanosensitivity of TRPA1 was attributed to a long stretch of ANK repeats, which were proposed to act as gating spring [53,54,55]. In mammals, TRPA1 expression has been confirmed in hair cell epithelia [54], but TRPA1 ablation failed to show auditory deficits [56,57]. Then again, experiments using isolated neurons [58,59], cell lines [60] and mechanosensitive sensory afferent fibers [61,62] imply TRPA1 in mechanosensation. Finally, TRPA1 was shown to contribute to the most abundant type of mechanically activated current, intermediately adapting macroscopic currents (IAMCs), with 43% reduction in small-diameter neurons from *trpa1* KO mice [63]. Other mechanically activated currents (such as Slow AMC and Rapid AMC) in small- and large-diameter neurons do not require TRPA1 for normal mechanosensory function [63]. This suggests a positive contribution of TRPA1 to mechanosensation in only a specific type of nociceptors. Additionally, increased expression of TRPA1 in the cellular membranes following acute activation might trigger chronic mechanical hypersensitivity. Nevertheless, it remains uncertain whether TRPA1 directly or indirectly contributes to mechanosensitivity.

**Figure 1:**
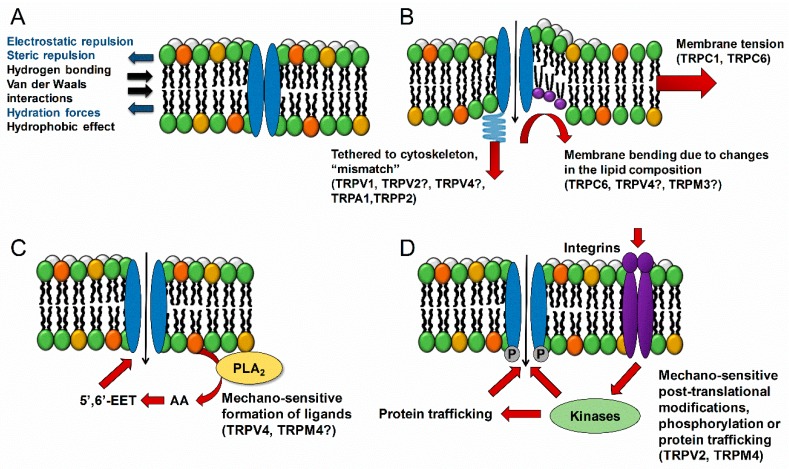
Mechanisms of mechanosensing. (**A**) All transmembrane channels are exposed to lateral forces from the bilayer creating lateral pressure acting on the channel. If the equilibrium is changed, conformational protein changes will occur modulating channel activity. (**B**) Changes in the membrane tension such as membrane ordering can induce channel gating [1]. The reorganization of the cytoskeleton due to mechanical stress might induce a mismatch between the conformation energy of a cytoskeletal tethered channel and the intrinsic lipid tension, causing the channel to open [1]. Changes in the membrane composition due to the insertion of specific lipids (phosphatidylcholines, lysophosphatidylcholines, or arachidonic acid) or lipophilic chemicals induce changes in the bilayer curvature or order and can lead to channel gating [1]. (**C**) Mechanical stimulation of the membrane can trigger the production of intracellular messengers, for instance the activation of PLA_2_ results in the formation of arachidonic acid, which by itself or by subsequent metabolization products activates the channel, as was shown to be the case for TRPV4 [39]. (**D**) Activation of accessory proteins such as integrins or kinases can regulate both the activity, trafficking and expression levels of the channel.

A question that arises is that of how mechanosensitive proteins detect changes in the membrane properties. Transmembrane proteins are exposed to negative and positive pressures produced by the bilayer. In normal conditions, the protein conformational energy matches the membrane energy profile. A change of that equilibrium, by membrane stretch or bending, results in modulation of the protein activity, as is the case for the ion channel conformational change and channel opening (Figure 1). This indicates that the insertion of lipids or membrane-modulating chemicals could induce channel gating. Other important factors involve accessory proteins for instance integrin clustering or cytoskeletal reorganization. Many TRPs are modulated by phospholipase C (PLC) activity, functionally linking them to GPCRs (e.g., Gq/11 linked to PLCβ) and tyrosine kinase receptors (via PLCγ). In addition, activity of TRP channels is modulated by intracellular Ca^2+^ changes and phosphorylation [64,65].

## 2. TRP Channel Modulation by the Local Lipid Environment

**Figure 2:**
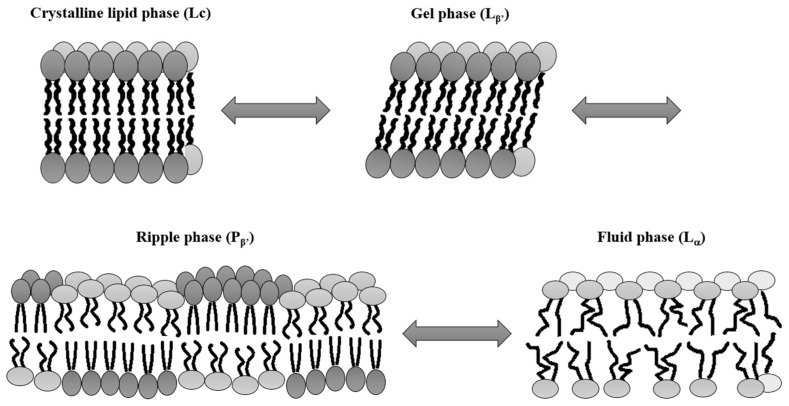
The lipid membrane phases. In the solid crystalline phase (L_c_), lipids are highly ordered into all-trans chain conformation generating a compact lipid environment which is characterized by a large reduction in lateral lipid diffusion and low levels of membrane hydration. Lipids are tightly packed parallel to each other into a hexagonal lattice with a spacing of roughly 0.4 nm [79]. The lipid hydrocarbons chains can be tilted with respect to the crystalline phase, thereby forming a gel phase (L_β’_) reliant on lipid composition and hydration levels or the presence of other ions. [73]. Depending on the composition of the bilayer, the transition between gel and fluid phase (L_α_) arises at a specific temperature called the thermotropic phase transition (Tm). This process is usually preceded by a so-called pre-transition inducing the formation of a ripple phase (P_β’_), characterized by periodic one-dimensional ripples. In the fluid phase, the lipids are highly disordered, and the triangular lattice order is lost with an increased lateral and rotational diffusion of the lipids (Modified from Heimburg [80]).

Biological membranes consist of a lipid bilayer and numerous proteins. This complex and dynamic system establishes a protective boundary around the cell’s interior [66]. Lipid bilayers are composed of two opposing leaflets made of multiple, asymmetrically distributed lipids species, belonging to the main three classes, including glycerophospholipids, sphingolipids and sterols [66,67,68]. Amphipathic lipids, consisting of polar, hydrophilic head groups and apolar, hydrophobic chains, tend to aggregate in the lipid-water systems into specific phases such as micelles, lamellar, cubic or inverse hexagonal phases [69,70]. This supramolecular phase organization is driven by the shape and concentration of the amphipathic molecule as well as the system’s temperature. The lipid phase organization reflects lipid packing at the lowest energy needed to balance repulsive head group interactions and hydrophobic chains effects. The most common lamellar, two-dimensional leaflets can exist in different states as crystalline phase (L_c_), gel phase (L_β’_), ripple phase (P_β’_) and fluid phase (L_α_) (Figure 2) [71,72,73,74].

Lateral organization of biological membranes is influenced by temperature, pH, the intercalation of chemicals or simply the presence of a different lipid species such as cholesterol. Cholesterol is a vital sterol component of most animal cell membranes, which is crucial for maintaining membrane integrity. High concentrations of cholesterol induce conformational ordering of the lipid chains followed by stiffening of the membrane, thereby acting as a permeability barrier [75]. In contrast, cholesterol depletion significantly increases the bilayer permeability, affects transport functions, membrane enzyme activities and the conformation of membrane proteins [76].

In addition, cholesterol determines membrane thickness and fluidity. While the cholesterol content in eukaryotic plasma membranes usually exceeds 20 wt%, it is non-homogenously distributed in the membranes of intracellular organelles, varying between 3 and 8 wt% [77]. This amphipathic molecule has a non-polar, hydrophobic planar hydrocarbon ring, and an isooctyl tail and a hydroxyl (OH) group making it soluble in water. This chemical structure renders specific interactions between cholesterol and other membrane components with the cholesterol backbone inserted in the bilayer hydrocarbon core and its OH group penetrating into the polar headgroup region.

Cholesterol might influence ion channel activity either by direct interaction with ion channels, modifying the mechanical properties of the plasma membrane or facilitating the formation of lipid-enriched microdomains known as lipid rafts [78]. These are small (10 to 100 nm) signaling platforms which are highly enriched in cholesterol and sphingolipids in the outer exoplasmic leaflet and associated with phospholipids and cholesterol of the inner cytoplasmic leaflet of the membrane [67].

The local lipid environment plays a crucial role in the expression pattern and function of several TRP channels that preferably segregated into cholesterol-rich lipid rafts. These include members of the TRPC subfamily (TRPC1, TRPC3, TRPC4 and TRPC5) [81,82,83,84], TRPV1 [85], TRPV4 [86], TRPM8 [87] or the *Drosophila* photoreceptor TRP-like channel (dTRPL). Interestingly, cholesterol regulates these channels in distinct manners. For instance, depletion of cholesterol reduces the activity of TRPV1 [85,88,89,90], TRPV4 [91] and dTRPL [92], but increases stimulation of TRPM8 [87] and TRPM3 [93]. Moreover, there is increasing evidence that TRPA1 activation maybe induced by changes in the lipid environment [94,95,96].

The TRP channels present in lipid rafts would be highly influenced by changes in bilayer rigidity, thickness, fluidity, and phase. Nevertheless, further investigation is needed to fully understand the nature and the mechanisms by which changes in the membrane phase state affect ion channel properties.

## 3. TRP Channel Modulation by the Conically-Shaped Lipid Diacylglycerol

**Figure 3:**
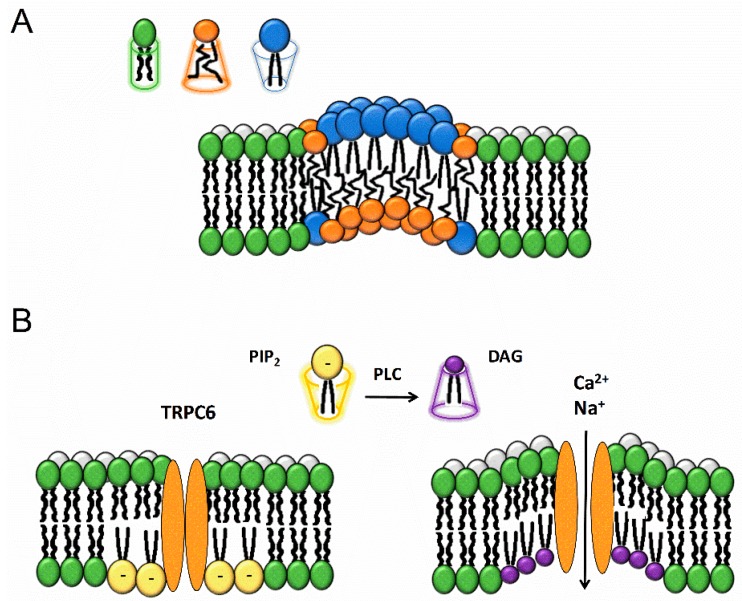
Lipids and membrane bending. (**A**) The membrane curvature can be altered by inserting lipids with specific shapes into the bilayer. Lipids can have a cylindrical (e.g., dioleoylphosphocholine), conical (e.g., dioleoylphosphotidylethanolamine, DAG and cholesterol) or inverse conical (e.g., lysophosphocholine) geometry [99]. Similar lipids tend to self-assemble in dynamic macrostructures depending on their lipid geometry and thus create asymmetric lipid bilayers. The increase in variability of pressure distribution along the membrane leaflets leads to different monolayer torques. The leaflet with the bigger torque bends to restore the system torque balance. (**B**) PLC-dependent breakdown of large-head, charged PIP_2_ to small-head, uncharged DAG occurs predominantly in the inner leaflet of the membrane in the close proximity to TRPC6. This causes inner leaflet deflection inducing changes in the curvature, resulting in bilayer stress and TRPC6 channel opening.

Biological membranes are asymmetric, with their individual leaflets having different properties that offer distinct microenvironments for different protein expression, trafficking and signaling processes. Furthermore, plasma membrane curvature defines the morphology of the cell and contributes to local membrane subdomains. The curvature-coupling hypothesis states that different lipids will induce specific local spontaneous curvatures (Figure 3A). Pure lipid bilayers stay flat, and upon stimulation can be curved towards the cytoplasm (positive curvature) or away from the cytoplasm (negative curvature) [97]. Fluctuations in one of the leaflets will induce changes in the second leaflet. For example, lipids with large, bulky heads and short chains will be preferentially present in the outer membrane while long chain, small heads lipids will be localized in the inner leaflet at the same position. Further, to achieve specific intrinsic curvature, membrane lipids are sorted in several ways. Lipids can be redistributed between opposing leaflets or between different regions, resulting in specific curvatures in distinct membrane regions [98].

The conically shaped lipid diacylglycerol (DAG) has been shown to directly activate members of the TRPC3,6,7 subfamily of the TRP channels, either added exogenously to cells or generated in response to PLC-coupled receptors (Figure 3B). TRPC6, is activated by mechanically or osmotically induced membrane stretch/deformation in a manner inhibited by the tarantula peptide toxin GsMTx-4, an inhibitor of mechanosensitive ion channels. Further, PLC-independent mechanical stress causing membrane thinning can also trigger TRPC6 activation. This mechanism directly depends on the lateral-lipid tension and lipid-protein mismatch, in such a way that the stretch-induced reduction in membrane bilayer thickness shifts the channel conformation to the open state [44]. In this sense, DAG might act similarly by changing membrane curvature, whereas GsMTx-4 may relieve membrane lipid stress and inhibit channel activation [44].

In addition, changes in the membrane curvature can be also induced by the intercalation of chemicals. For instance, trinitrophenol is a negatively charged amphiphilic compound that accumulates in the outer leaflet of the plasma membrane, leading to crenation. Another chemical, chlorpromazine, inserts into the inner leaflet of the membrane and induces cup formation. In this regard, trinitrophenol has been shown to activate TRPA1, whereas chlorpromazine inhibits it, suggesting that this channel is sensitive to membrane deformation [94].

## 4. TRP Channel Modulation by the Natural Membrane Modulator Lipopolysaccharide

Lipopolysaccharides (LPS) are essential structural components of the outer membrane of gram-negative bacteria [100,101]. Due to their vital role in the bacterial growth and survival, endotoxins structures are highly conserved within all gram-negative species [102]. Either as an isolated compound or as part of the bacterial outer membrane, endotoxins play a crucial role in the pathogenesis and onset of symptoms of gram-negative infections. LPS are one of the most potent mediators inducing local or generalized inflammatory responses in humans, animals and even plants [103].

**Figure 4:**
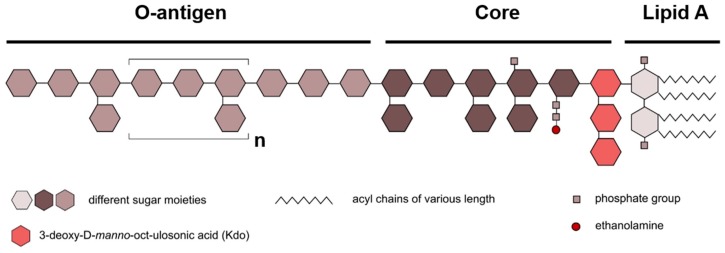
Schematic diagram of the general LPS structure. Lipopolysaccharides consist of three main subunits: lipid A, the core region and the O-antigen. The number and chemical structure of the acyl chains can vary. Sugar moieties are depicted as hexagons in different colors. The number and chemical nature of these sugars can differ (Modified from Steimle et al. [102]).

LPS from all gram-negative bacteria consist of three chemically distinct domains: the acylated and phosphorylated lipid A, the core oligosaccharide, and the O-antigen or O-specific polysaccharide (Figure 4). The O-antigen forms the outermost part of the LPS and is a major target for the host immune system. It consists of repeating sugar units (O-units), containing two to eight sugar residues. The types of sugar, the sequence and chemical linkage varies within and between O-units. For this reason, the O-antigen differs tremendously among bacterial strains [104]. The core oligosaccharide is an often-branched and phosphorylated hetero-oligosaccharide composed of up to 15 sugars and is divided to an inner and outer core. The inner core, proximal to the lipid A, contains residues of keto-deoxyoctulosonate (kDo) and heptose. Generally, the inner core tends to be well conserved, reflecting its importance for bacterial outer membrane integrity. The outer core is more variable, consistent with regions exposed to selective pressures of the environment (e.g., host responses). It typically accommodates several hexose residues such as glucose, mannose and galactose [105].

**Figure 5:**
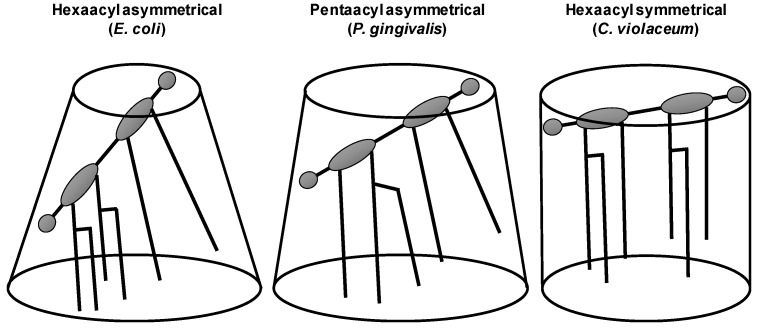
LPS macrostructure proposed for different lipid A moieties. (Modified from Seydel et al. [110]).

LPS and lipid A are amphiphilic molecules. The chemical variations of lipid A strongly influence its capacity to interact and activate receptors of the immune system. The intrinsic conformations have been demonstrated to be responsible for its agonistic and antagonistic activity [106]. Lipid A is responsible for the toxic effects associated with gram-negative bacteria infections [107]. It constitutes the minimal structural moiety required to trigger the activation of the canonical Toll-Like Receptor 4 (TLR4)-mediated pathway [108,109]. Usually lipid A is composed of a glucosamine disaccharide backbone attached to up to four fatty acid residues, which can be further substituted to provide up to seven acyl substituents. The number, position and length of these acyl chains and the presence of charged groups on the polar heads as well as the number of phosphate groups coupled to the disaccharide backbone can differ. Each glucosamine can carry the same number of acyl chains denoting it a ‘symmetrically’ acetylated lipid A. *E. coli* LPS is an example of ‘asymmetrically’ acetylated lipid A, since four of its six acyl chains are carried by one of the glucosamines [102,105]. Despite its general structural conservation, lipid A structures display considerable micro-heterogeneity among gram-negative bacterial species, directly resulting in altered endotoxic strength [103]. The bioactivity of lipid A is related to its three-dimensional shape and the biochemical composition (i.e., the chemical structure, the net charge and the inclination of the head group) [106]. Endotoxically active lipid A can be distinguished from endotoxically inactive lipid A (with antagonistic properties) by the intrinsic shape with a larger inclination of the diglucosamine ring plane with respect to the membrane surface and, concomitantly, a conical instead of a cylindrical shape [110,111] (Figure 5). For example, the hexa-acylated *E. coli* lipid A acts as a potent agonist for all mammalian cells.

The detection of LPS leads to a strong host immune system activation. Classical immune pathways involve LPS recognition by pattern recognition receptors (PRRs) [112], of which the most studied is the TLR4 pathway [109]. Activation of PRRs triggers signaling cascades inducing the biosynthesis of inflammatory cytokines (TNF-α, IL-1 and IL-6), as well as ROS and antimicrobial peptides [109]. In addition to the classical TLR4-mediated pathway, other proteins have been shown to be involved in LPS recognition and bioactivity [113,114,115]. For example, LPS activates multiple TRP cation channels including TRPA1 [95], TRPV1 and TRPV4 [116] in a TLR4-independent manner. TRPA1 activity has been related to several acute LPS responses in mice such as hyperalgesia, edema, release of calcitonin gene-related peptide (CGRP) and arterial dilation establishing channel function in triggering acute inflammatory and pain responses [95]. TRPA1 activation by bacterial endotoxins produces avoidance during feeding and oviposition in *Drosophila melanogaster* [117]. Further, TRPV4 activation in airway epithelial cells induces protective responses such as nitric oxide production and increased ciliary beat frequency [116]. Thus, TRP channels are sensors of LPS, operating independently of classical TLR4 signaling.

**Figure 6:**
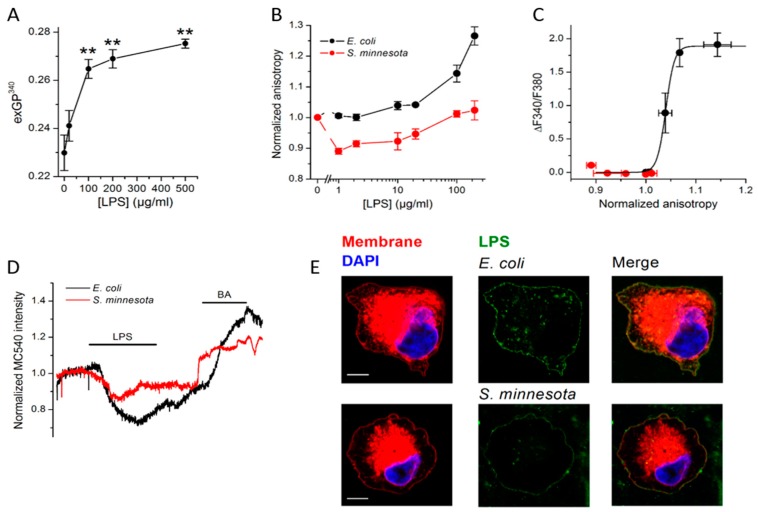
Effect of LPS insertion on membrane properties. (**A**) Determination of Laurdan excitation Generalized Polarization (exGP) is a useful tool in describing membrane fluidity as this probe displays a shift in the emission when in contact with ordered or disordered membranes [136,137]. The GP values recorded in one component lipid giant unilamellar vesicles (GUV) are between −0.3 to 0.3 corresponding to the fluid and between 0.5 and 0.6 in gel phase [138]. The exGP values determined in DPPC GUVs indicate membrane rigidification induced by *E. coli* LPS. (**B**) Fluorescence anisotropy of membrane sensitive dye diphenylhexatriene (DPH) changes upon alterations of the membrane fluidity. Intercalation of *E. coli* LPS into CHO-mTRPA1 membranes caused anisotropy increase demonstrating reduction in its fluidity. Contrary, *S. minnesota* LPS that did not prompt significant changes. (**C**) Relation between LPS-induced increases in intracellular Ca^2+^ levels caused by TRPA1 activation and change in DPH fluorescence anisotropy recorded in CHO-TRPA1 cells upon application of *E. coli* or *S. minnesota* LPS (0–100 µg/mL), clearly indicted differential effects of both LPS. (**D**) Merocyanine 540 (MC540), a lipophilic, viscosity probe which fluorescence increases upon binding to membranes in fluid-phase, has been used to further differentiate between effects induced by *E. coli* or *S. minnesota* LPS. Benzyl alcohol (BA), a well-described membrane fluidizer [139], has been used after LPS wash out. *S. minnesota* LPS prompted weaker effects in the plasma membrane mechanical properties that could be attributed to a lower efficiency of its insertion into the cellular membranes (**E**). Representative Airyscan confocal images of CHO-TRPA1 cells stained with membrane mask (red), DAPI (blue) and treated with Alexa Fluor 488 LPS (green) from *E. coli* (20 µg/mL top panel) or *S. minnesota* (20 µg/mL bottom panel). Scale bar, 5 µm. Reproduced with permission from Startek et al. [96].

Yet, the mechanism of activation by LPS remained elusive. Interestingly, the most sensitive TRP channel for LPS, TRPA1, plays a role in mechanosensation [62,118] and can be activated by chemicals, which are known to alter membrane properties [94]. A recent study demonstrated that mechanical perturbations of the cellular membrane induced by *E. coli* LPS can trigger TRPA1 activation both in cellular and artificial membranes (Figure 6 A–D) [96]. These findings offer decisive support to previous reports on the ordering of lipid membranes by *E. coli* LPS [119,120,121,122,123]. LPS was shown to insert into 1,2-dimyristoyl-*sn*-glycero-3-phosphocholine (DMPC) bilayers, prompting significant reduction of the elastic strength, subsequently reducing vesicles size [124].

*E. coli* LPS intercalation into the membrane of red blood cells caused volume decrease, increased membrane viscosity, as well as reduction of stress tolerance [120,121,122]. Similarly, *E. coli* LPS insertion into hepatocyte membranes produced an increased viscosity and delay in the membrane fluidization induced by heat [123]. Further, it was demonstrated that LPSs with different conformations of lipid A, such as LPS from *S. minnesota*, are less effective in inducing changes in the membrane order. The reduced membrane insertion efficiency of *S. minnesota* LPS compared to *E. coli* LPS (Figure 6E) indicates the importance of the shape of lipophilic lipid A [96]. These findings suggest that the conically-shaped lipid A moiety of *E. coli* alters membrane properties to a greater extent than the cylindrical ones such as those from *S. minnesota*. Further support for this idea came from the correlation between the lipid A shape and the ability of different LPS molecules to activate TRPA1 *in vitro* and to cause inflammation *in vivo* [95]. These effects follow studies reporting lower bioactivity of *S. minnesota* lipid A with respect to *E. coli* lipid A on TRPA1 channel activation and human-derived cells [95,125]. As the TRPA1 subunit organization in the cellular membranes indicates a large contact surface with membrane lipids [126], changes in the physical properties of the membrane such as order, tension, thickness and curvature resulting from LPS intercalation in the vicinity to the channel could alter the TRPA1-bilayer interaction and induce channel opening [127].

It should be noted that the *E*. *coli* LPS concentrations shown to activate TRPA1 (EC_50_ ~3 µg/mL) [95] are about 2 orders of magnitude higher than those activating the canonical TLR4 pathway *in vitro*. However, it was reported that during pathological conditions such as severe endotoxemia, plasma LPS levels can rise into the µg/mL range [128]. Furthermore, relatively high LPS concentrations are present in urine and locally infected tissues [129] and are used in current experimental models of endotoxin challenge in LPS-evoked pain behavior experiments [130] and LPS-induced airway inflammation [131,132,133]. Bacterial lysis during acute infection can also result in locally high concentrations especially in the very small volumes of acute dental abscesses and micrometer-thin mucosal layers at epithelial barriers.

Further, fever triggered by the stimulation of the immune system and local changes of temperature induced by neurogenic inflammation at the site of infection may influence the response of TRP channels to LPS. However, the outcome of these interactions is difficult to predict, because these channels are also strongly modulated by temperature. Although we recently reported that LPS-induced activation of TRPM8 by LPS was enhanced at cold temperatures [134], the underlying mechanism of this effect and the existence of such interactions for other sensory TRP channels (TRPA1, TRPV1, TRPV4 and TRPM3) remain unexplored.

As LPS insertion produces lateral membrane compression, the mechanism underlying TRPA1 activation by LPS is different from that operating in “classical” mechanosensitive channels e.g., membrane stretch [1,10,135]. Elucidation of TRPA1 activation mechanisms by LPS helps to understand novel protective defense mechanisms against microbial species where TRP channels activation triggers protective responses mediated by sensory neurons.

## 5. TRP Channel Modulation by Lipophilic Compounds 

Local anesthetics (LAs) inhibit voltage-gated Na^+^ channels (VGSCs), thereby preventing the generation of neuronal action potentials and pain sensation [140,141]. Many widely used LAs, such as procaine, lidocaine and tetracaine interact with the membrane hydrophobic region and can alter its properties, resulting in the modulation of specific signaling events. It has been suggested that interactions between LAs and the membrane leads to changes in the bilayer bending rigidity and tension [142,143]. LAs were reported to rearrange the intermolecular hydrogen-bonded network among phospholipids and alter the orientation of their P–N dipole, inducing an increase in membrane fluidity [144]. VGSCs are expressed in lipid rafts and high cholesterol levels have been shown to promote LAs partitioning into bilayers [145]. Further, the artificial raft-mimicking phases are destabilized by inserting LAs [143]. Thus, if LAs destabilize specific membrane domains, nearby VGSCs may also be affected. Once lipid rafts are destabilized, the order, structure, and movement of the VGSC subunits forming the structure of the channel gates may be altered [146]. Different LAs have different effects on the membrane, as for instance, tetracaine induces higher miscibility temperature, line tension at the ordered/disordered phase interface, and lipid main transition temperature and phase separation than lidocaine [147]. Additionally, other molecules such as n-alcohol general anesthetics are reported to correlate increasing molecule hydrophobicity, producing a decrease in lipid transition temperature, with an increase in the strength of anesthesia [148,149].

Interestingly, in high concentrations, LAs have excitatory effects attributed to activation of TRPV1 and to a lesser extent of TRPA1 channels, resulting in a Ca^2+^-dependent release of CGRP and substance P [150,151,152]. This leads to pain, local tissue damage, inflammation and neurotoxicity [153]. Lidocaine not only activates both rodent TRPV1 and TRPA1 channels, but also inhibits them in a concentration-dependent manner [150]. Although the mechanism underlying TRP activation by LAs may involve changes in the membrane fluidity, binding to specific channels domains cannot be excluded. Activation of TRPV1 by lidocaine was reported to involve interactions with a vanilloid-like binding domain composed of amino acid residues located in TM3 and TM4 and the respective cytosolic interfaces [151]. It also requires an interaction of PIP_2_ with the proximal C-terminal TRP domain [151]. Capsazepine, the competitive TRPV1 antagonist, prevented activation of TRPV1 by lidocaine [151]. Lidocaine induced TRPV1 desensitization involves channel dephosphorylation through a mechanism involving calcineurin. Desensitization could be also reduced by PKA- and PKC-mediated phosphorylation and Ca^2+^-dependent inactivation involving calmodulin [151]. On the other hand, activation of TRPA1 by LAs does not involve either vanilloid-binding domain or a TRP domain. LAs were shown to interact with TM5 of TRPA1, which may explain the differences between rodent and human TRPA1 lidocaine-evoked currents [150]. Nevertheless, lidocaine activates the menthol-insensitive mutant mTRPA1-S876V/T877L [150,154]. The mechanism of TRPA1 activation may also involve direct stimulation by PLC and regulation by PIP_2_ [150]. Further, general anesthetics such as desflurane, isoflurane, sevoflurane and halothane were suggested to act in a membrane-delimited fashion. However, TRPA1 activation by these compounds did not correspond with their ability to partition into cellular membranes, arguing against induction of changes in the membrane fluidity as underlying mechanism of channel activation [155]. Furthermore, the response cutoff correlated with 8–10 carbon length chain of alcohols, indicating existence of a binding pocket within TRPA1 [155].

Another group of chemicals, primary alcohols, are TRPA1 activators inducing skin, eye and nasal irritation [156,157]. Potentiation of harmful effects and activation of TRPA1 was correlated with the increase in the length of a carbon chain (from C2OH to C8OH) [158]. The comparison between primary and secondary alcohols with different lengths of the carbon chains revealed that TRPA1 activation ability depends mainly on the length of the carbon chain and not on the position of their hydroxyl group [158]. These results strongly suggest that the octanol/water partition coefficient (lipophilicity) may be a critical determinant of TRPA1 activation. Similar effects were also reported for other TRP channels, e.g., TRPV1 [159,160], indicating a possible general activation mechanism.

Notably, a very large group of non-electrophilic TRPA1 agonists has, as a common feature, the ability to intercalate into cellular membranes, inducing mechanical alterations. The analysis of the chemical structures and properties of thymol and different alkyl-substituted phenol derivatives suggests for a correlation between the partition coefficient (logP) and the potency to activate the channel [161]. Similar conclusions could be made for a series of parahydroxybenzoates (parabens), for which an increase in calculated logP value correlated with a reduced EC_50_ value for TRPA1 activation [162]. Also, the lipophilic farnesyl thiosalicylic acid and its analogs [163] and 6-gingerol analogs [164,165] are able to activate TRPA1. Similarly, the highly lipophilic compounds leucettamols A and B and of their semi-synthetic analogues were shown to modulate TRPA1 activity [166]. Despite the increasing evidence that TRPA1 activation by lipophilic chemicals could be induced by changes in the channel local lipid environment caused by the compounds intercalation, the full mechanism of channel activation remains elusive.

Moreover, cells undergo changes in membrane composition and physical properties that tend to preserve specific membrane order in response to harmful environmental chemicals, such as crude oil [167], PCB-153 [168], hydrocarbons, alcohols and detergents [169]. The effects of temperature on the composition of the biological membranes are also well described in plants [170], poikilotherms [171], Archaea [172], zooplankton [173], fish [174,175] and mammals [176,177]. One of the mechanisms contributing to the regulation of cellular membrane order, known as homeoviscous adaptation (HVA), is based on the stabilization of membrane fluidity by changing the relative concentrations of saturated and unsaturated membrane fatty acids. Although originally the HVA theory was formulated for a temperature-dependent response in bacteria, it has been later shown to have a much broader evolutionary significance [176,178]. For instance, mammalian membrane order is preserved by biological membranes in response to hydrostatic and osmotic pressure [174,179], low magnetic field strength [180], chemicals [168] and lipids such as cholesterol content. Membrane fluidization by exogenous chemicals or heat [181,182] increases the concentration of intracellular Ca^2+^, which may act as a second messenger in the cellular pathways (e.g., activation of heat-shock proteins [183]). Because sensory TRPs can be activated by a wide variety of external stimuli, we hypothesize that these channels may be involved in the mechanisms underlying the remodeling of cellular membranes upon chemical and thermal challenge. In a broader context, cellular phospholipid homeostasis could help to understand pathophysiology of multiple diseases such as cancer [184], Alzheimer’s disease [185], liver disease [186,187] and diabetes [188] in which fine balance between in membrane properties is disturbed. 

Taken together, evidence recently accumulated led us to conclude that the “mechanical detection” of chemical partitioning in cellular membranes by TRP channels might be part of a fundamental mechanism in which TRP channel activation by chemical and physical stimuli might result not only from classical compound-receptor interaction but also from their mechanosensory properties [15,94].
